# White matter microstructural characteristics in newly diagnosed Parkinson’s disease: An unbiased whole-brain study

**DOI:** 10.1038/srep35601

**Published:** 2016-10-20

**Authors:** Ming-Ching Wen, Hannah S. E. Heng, Samuel Y. E. Ng, Louis C. S. Tan, Ling Ling Chan, Eng King Tan

**Affiliations:** 1 Department of Research, National Neuroscience Institute, Singapore; 2 Department of Neurology, National Neuroscience Institute, Singapore; 3 Duke-NUS Graduate Medical School, Singapore; 4 Department of Diagnostic Radiology, Singapore General Hospital, Singapore

## Abstract

Parkinson’s disease (PD) is a debilitating neurodegenerative disorder. Findings on specific white matter (WM) alterations in PD have been inconsistent. We hypothesized that WM changes occur in early PD patients and unbiased whole-brain analysis may provide additional evidence of pathological WM changes in PD. In this study, we examined various indexes of WM microstructure in newly diagnosed PD patients at the whole-brain level. 64 PDs with Hoehn & Yahr stage 1 (HY1PDs), 87 PDs with Hoehn & Yahr stage 2 (HYPD2s), and 60 controls (HCs) were recruited. Tract-based spatial statistics (TBSS) and diffusion connectometry were used to identify changes of WM pathways associated with PD. There were no significant differences in axial diffusivity, but HY1PDs exhibited greater fractional anisotropy (FA) and decreased mean and radial diffusivities (MD and RD) in callosal, projection, and association fibres than HCs and HY2PDs. Motor severity was inversely correlated with FA, but positively correlated with MD and RD in PD patients. Connectometry analysis also revealed increased WM density in the aforementioned tracts in PD patients, compared with HCs. Our study reveals WM enhancement, suggesting neural compensations in early PD. Longitudinal follow-up studies are warranted to identify the trajectory of WM changes alongside the progression of PD.

Parkinson’s disease (PD) is a progressive neurodegenerative disease histopathologically characterised by loss of dopamine neurons in the substantia nigra (SN) pars compacta. Diffusion tensor imaging (DTI) is a non-invasive neuroimaging technique that can encode information about the orientation of water molecular movements within the brain white matter (WM) tracts. This technique has been widely adopted to study pathological changes in the WM of patients with various neurodegenerative diseases, including PD^1^. The overall diffusivity and the extent of diffusional directionality can be quantified by mean diffusivity (MD) and fractional anisotropy (FA)^2^. Apart from quantification of MD and FA, axial diffusivity (AD) and radial diffusivity (RD), referring to the diffusivity along the axon and perpendicular to the axon, have shown greater specificity to underlying axonal and myelin alterations^3^.

Previous studies have demonstrated conflicting observations, such as decreased, increased, and unaltered FA of the SN being reported in PD^4^,^5^,^6^,^7^. Although two previous meta-analysis studies have shown decreased FA and unaltered MD of SN in PD patients vs. controls^1^,^6^, the sample sizes of the included studies were small and one showed a very high level of heterogeneity^6^. Prior DTI works in PD have mainly focused on FA with some additionally reporting one of the diffusivity measures, and predominantly used region-of-interest (ROI) methods to find difference in WM pathways of the SN^1^,^6^,^8^,^9^. There is increasing evidence to suggest that regions beyond SN may also be affected^10^,^11^,^12^. Hence unbiased whole-brain analysis may provide additional evidence of pathological WM changes in PD.

Recently, diffusion connectometry was developed to track the difference in voxels that have substantial association with the studied variables using density of diffusing spins, and can be tailored to study patient group data against a control population to identify the affected WM segments of the WM pathway^13^. This method, though complementary to the aforementioned existing analytic approaches, has not been used to study WM changes associated with PD pathology.

In addition, as the contrast in DTI comes from the microscopic random motion of water molecules in brain tissues, head motion can be a significant confounding factor to FA and diffusivity measures and lead to the observation of a decrease FA and increases in diffusivities, according to previous investigations^14^,^15^. Despite the importance of taking head motion into account in PD studies where patients might exhibit more head motion than healthy controls (HCs), most previously published studies have not clearly addressed how this issue was resolved.

In the current study, we examined different WM microstructural features of PD pathology in a large cohort involving drug naïve early and non-demented PD patients with less than two-year disease duration in comparison with healthy controls. We hypothesized that WM microstructural changes could occur in the early stages of PD and WM changes may be associated with the severity of motor symptoms when head motion is controlled.

## Results

### Demographic and Clinical Findings

In total, this study included 211 subjects (60 HCs and 151 PDs) recruited from 11 different centres with good quality baseline DTI data. Among the 151 PDs, 64 were in Hoehn & Yahr stage 1 (termed HY1PDs) and 87 were in stage 2 (termed HY2PDs). There was no significant group difference in recruiting sites (Fisher’s exact test = 8.12, p = 0.62). None of the participants had the diagnosis of dementia at baseline. For PD patients, the mean disease duration was 6.77 months (SD = 7.03) and was comparable between HY1PDs and HY2PDs (t = −1.63, p = 0.11). Demographic and clinical data are presented in Table 1. There were no significant group differences in age (F = 0.83, p = 0.44), gender distribution (*χ*^2^ = 0.19, p = 0.91), handedness (Fiher’s Exact = 5.94, p = 0.20), or years of education (F = 1.17, p = 0.31). There were also no significant differences when comparing general cognitive function (F = 2.13, p = 0.12), depression (F = 0.03, p = 0.98), and head motion (translation: F = 1.19, p = 0.31, rotation: F = 0.88, p = 0.42). As expected, the PD group scored higher than the HC group on part III of the Movement Disorders Society (MDS) Unified Parkinson’s Disease Rating Scale (UPDRS-III^16^, [F = 260.62, p < 0.001]). Post-hoc analysis indicated that compared with HCs, HY2PD patients had greatest motor severity, followed by HY1PD patients.

### TBSS Analysis

**Figure 1:**
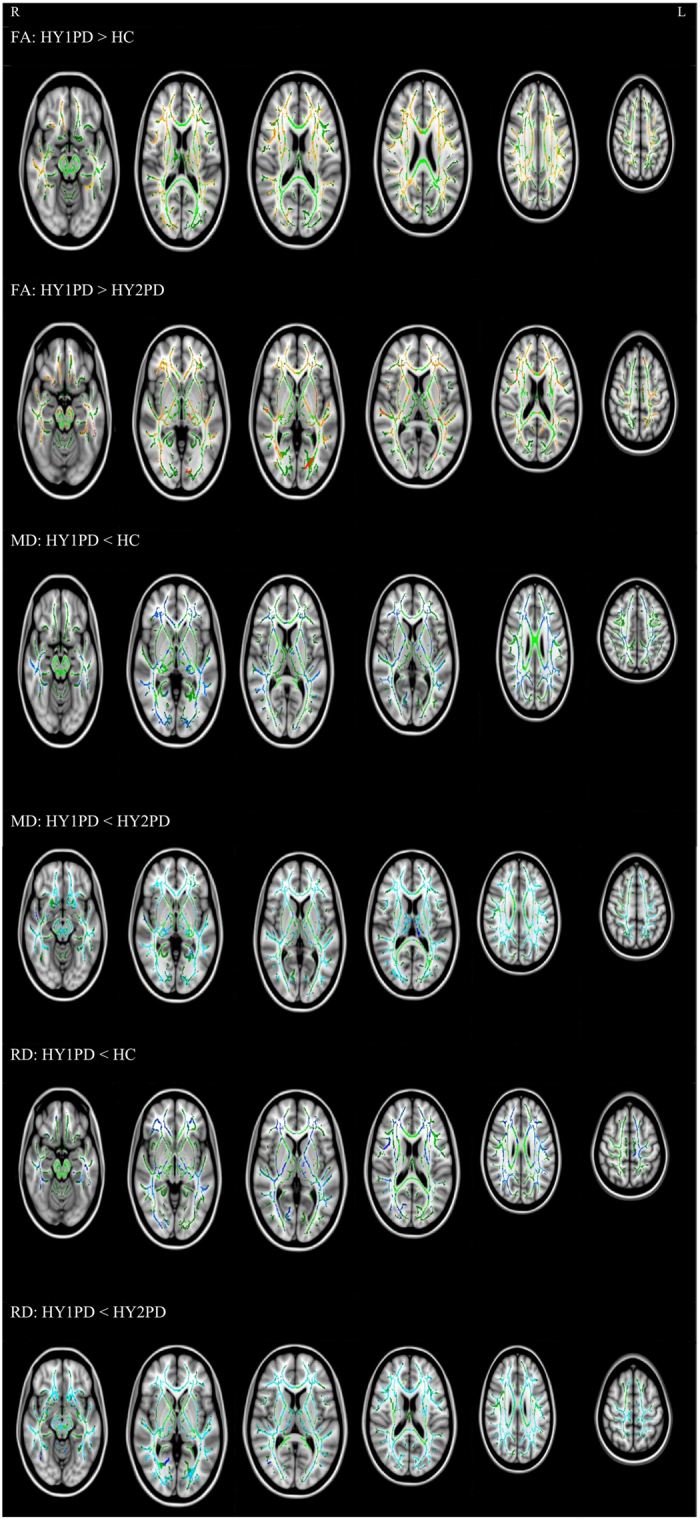
Group differences in FA. TBSS map showing areas of increased FA (red-yellow), decreased MD and RD (blue-light blue) in the WM tracts in HY1PD overlaid on the study-specific FA skeleton (green), compared with HC and HY2PD. All results are at p < 0.01, corrected for multiple comparisons using TFCE.

HY1PD patients showed increased FA in the corpus callosum, forceps minor, some projection fibres, including the thalamic radiation, anterior and superior corona radiata, and posterior internal capsule, and some association fibres, such as the external capsule, superior and inferior longitudinal fasciculi, inferior fronto-occipital fasciculi, and cingulum, compared with HCs and HY2PD patients. Conversely, HY1PD patients showed decreased MD and RD in the aforementioned WM tracts as opposed to HCs and HY2PDs. Figure 1 presents the WM tracts showing significant group differences in FA, MD, and RD. However, there were no WM tracts indicating significant group difference in AD. Compared with HCs, HY2PD patients did not show any difference in any of the DTI indexes.

**Figure 2:**
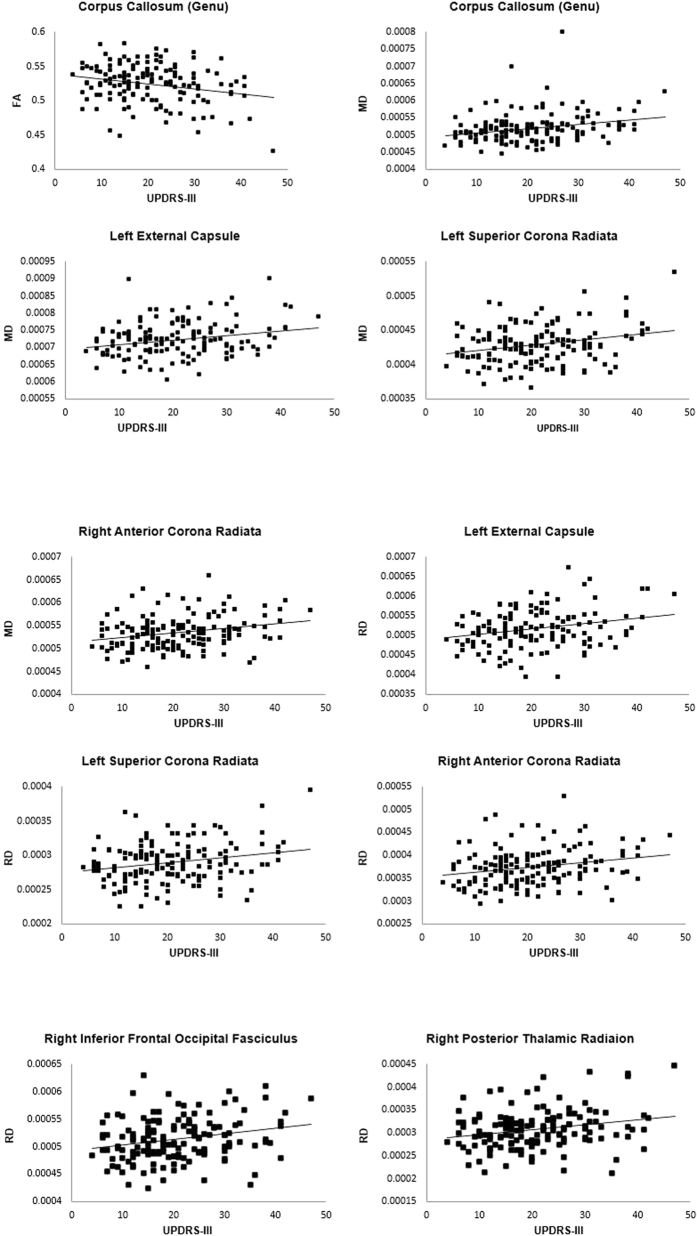
Scatterplots of WM tracts showing inverse relationships between UPDRS-III and FA and positive relationships between UPDRS-III and MD and RD in PD.

Correlating FA, MD, and RD values of these significant tracts with the severity of motor symptoms in PD patients revealed that motor severity was inversely correlated with FA of the genu of corpus callosum (r = −0.23, p = 0.042), but positively correlated with MD of the genus of corpus callosum (r = 0.26, p = 0.022), left external capsule (r = 0.21, p = 0.022), left superior corona radiata and right anterior corona radiata (rs = 0.25, ps = 0.022). Motor severity was also positively correlated with RD of the left external capsule and superior corona radiata (rs = 0.24 and 0.23, respectively, ps = 0.04), and right anterior corona radiata (r = 0.24, p = 0.04), inferior fronto-occipital fasciculus (r = 0.23, p = 0.04), and posterior thalamic radiation (r = 0.24, p = 0.04). Figure 2 shows the scatterplots of these significant correlations.

### Connectometry Analysis

**Figure 3:**
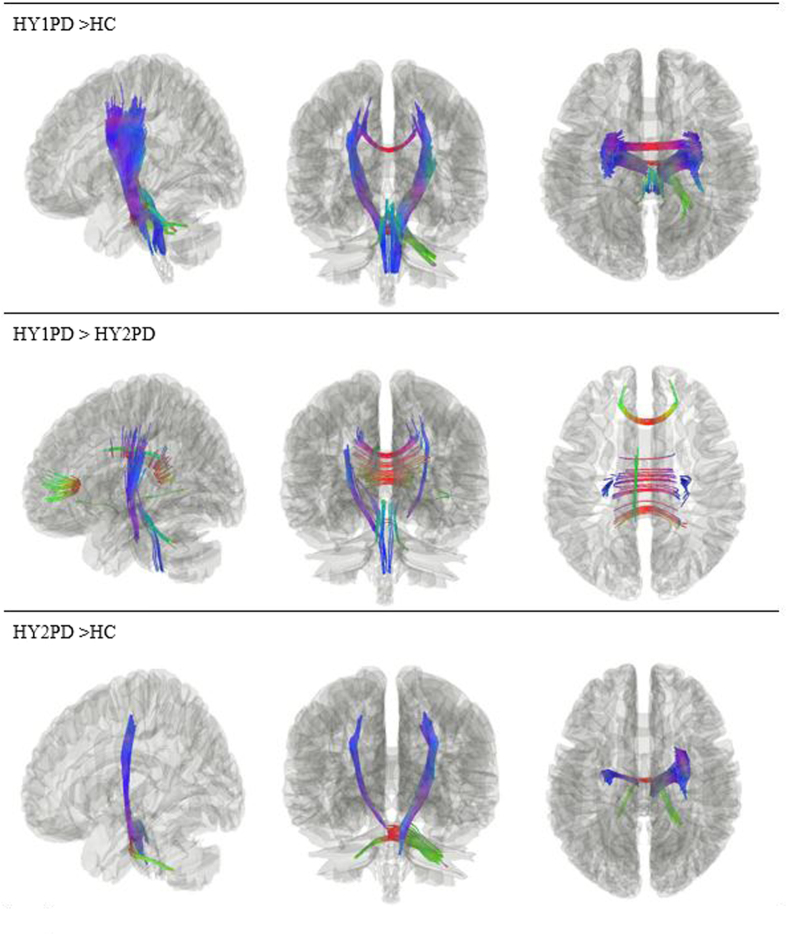
Connectometry results showing increased WM density in specific tracts of HY1PD patients compared with HCs and HY2PD patients and in tract of HY2PD patients compared with HCs (p < 0.05, FDR corrected for multiple comparisons).

The density of diffusion spins at various orientations was calculated for group comparisons. The results showed increased WM density of the corpus callosum, bilateral external capsule and cortico-thalamic tracts and right corticospinal tract in the HY1PD group as opposed to the HC group (p = 0.007). HY1PD patients also showed increased WM density of the corpus callosum, and bilateral external capsule, when compared with HY2PD patients (p = 0.008). Furthermore, compared with HCs, HY2PD patients had increased WM density in the cortical U fibre, bilateral external capsule, right cortico-thalamic tract and inferior fronto-occipital fasciculus, corpus callosum, and middle cerebellar peduncle (p = 0.0177). Figure 3 presents these significant WM tracts. In contrast, despite group differences were found in these tracts, none of them showed a significant correlation with the severity of motor symptoms (p > 0.05, FDR corrected).

## Discussion

This is one of the largest DTI studies on newly diagnosed early PD. We used two different whole-brain analytical approaches to unbiasedly examine WM tracts that significantly differentiated newly diagnosed PD patients in different disease stages from HCs and to explore the relationships of these WM characteristics with motor severity. No statistically significant alterations of AD were found among patients in the early stages of PD and with no more than 2-year disease duration. HY1PD patients, however, were found to have significantly increased FA, but decreased MD and RD in several projection and association WM tracts^17^ as well as callosal WM tracts (corpus callosum and forceps minor)^18^, compared with HCs and HY2PDs. Similarly, we observed significantly increased density (i.e., greater connectivity between adjacent voxels within a WM bundle) in the callosal, association and projection tracts and tracts in the brainstem of the PD groups as opposed to the HC group and of the HY1PD group as opposed to the HY2PD group. In addition, an inverse though weak correlation was noted between FA and motor severity, whilst positive and weak correlations between MD and RD and motor severity were found among PD patients.

Findings from our current work seem contradictory to the findings from two previous meta-analysis studies^1^,^6^ and two recent studies^8^,^9^ that decreased FA was mainly observed in the SN in PD patients, compared with HCs^1^,^6^. Notably, several more recent cross-sectional and longitudinal studies also found increased FA in the SN^4^,^7^ as well as tracts in the motor pathways, such as the corticospinal tracts^19^, or unaltered FA in PD^20^, compared with controls. Methodological variance, however, is likely to have contributed to these discrepant findings. Only ROI–based methods focusing on a small number of a priori brain regions were applied in these reports (except^20^), whilst both analytical methods in our current work were whole-brain based. Nonetheless, FA is usually considered an indication of WM integrity^2^, and MD and RD are indicative of loss of directional water molecular movement in WM tracts^21^ and myeline damage^22^,^23^. Unlike the study by Duncan and colleagues^20^, we noticed decreases of MD and RD in the tracts outside the SN and also elevated FA of these tracts in HY1PD patients. The discrepant findings may be due to different motor and cognitive characteristics of patients. In their study, PD patients were slightly more advanced (maximum H&Y = 3) with worse cognitive function as compared to HCs, whilst we had PDs in the earlier stages (maximum H&Y 2) with comparable cognitive function relative to HCs. With not only motor but cognitive impairments, it is likely to observe more enhanced incoherence of water movements within WM tracts.

Notably, the alterations of DTI indexes only exhibited in the HY1PD group, but not in the HY2PD group. Therefore, the increase of FA together with decrease of MD and RD shown in HY1PD may suggest neural compensations for the loss of dopaminergic input from the SN in the very early disease stage. This is in line with the view proposed by Brotchie and Fitzer-Attas^24^ that one manifestation of the compensation for dopaminergic deficiency may be a structural remodelling of neural circuitry^24^. As we also found motor severity being inversely correlated with FA and positively correlated with MD and RD, the view of neural compensations via the enhancement of FA and the reductions of MD and RD could be supported^24^. It is possible that as symptoms worsen, more neural resources may be reduced and the compensatory effects may diminish, in which case decreased FA or increased diffusivities might be expected. Moreover, our findings that patients in a slightly more advanced disease stage (i.e., HY2PD) had similar FA, MD and RD, and relatively little differences in WM density, compared with HCs, may reflect the attenuation of neural compensation. In other words, while neural compensation was observed in the HY1PD stage, it dissipated when disease progressed to the HY2PD stage. Nonetheless, studies with longer follow-up duration are needed to test the speculation of emergence and dissipation of neural compensation in the progression of PD.

Increased WM density was found in the brainstem tracts as well as the callosal, association and projection tracts in the PD groups as opposed to the HC group and in the HY1PD group relative to the HY2PD group. This is in line with a previous study showing elevated WM tract density in brainstem and extrapyramidal networks of cognitively normal PD patients^25^. Connectometry analysis further identified WM differences between HY2PDs and HCs, albeit to a lesser extent, compared with the difference between HY1PDs and HCs. Enhanced density of HY2PD was not detected using TBSS. The common advantage between TBSS and connectometry is that both methods have been claimed to reduce partial volume artefacts, despite employing different analytical operations^26^,^27^. Although FA, MD, and RD are useful indicators of WM healthy or pathological conditions, substantial evidence has indicated its susceptibility to partial volume effect^28^,^29^,^30^. In comparison, diffusion connectometry uses the density of diffusion spins derived from a model-free approach, as the core diffusion measurement to reveal the compactness of fibre bundles^13^,^27^. The current study is the first to apply this method to examine PD pathology. Unlike ROI and TBSS methods which assess discrete regions in the brain, connectometry was proposed to firstly, measure the degree of connectivity between adjacent voxels within a WM fibre defined by the density of the diffusing spins and, secondly track only the segment of fibre bundle in the entire brain that exhibits significant association with the study variable^13^. This approach therefore provides complementary information about WM characteristics associated with PD pathology. Our connectometry findings supported increased FA, but decreased MD and RD in the external capsule, corpus callosum, and cortico-thalamic tracts which were also found from the TBSS analysis. Increased WM integrity and connectivity in early PD demonstrated in our study do not appear to follow the early neuropathological features in Braak *et al*.’s post-mortem staging^31^. However, there is a lack of evidence of a direct relationship between Braak staging and the clinical severity of PD^32^.

Although prior work has demonstrated that head motion may affect the DTI results, depending on the movement direction and pattern^14^,^15^, most DTI studies in PD did not specify how this artefact was controlled. On the contrary, our study only included participants with minimum head motion and we further ensured that the PD group did not substantially have more movements during the acquisition of DTI, compared with HCs. Other strengths of our study include having a large cohort of demographically and cognitively well-matched HCs and PDs. The PD patients were newly diagnosed with limited disease duration and were drug naïve. This patient composition thus allowed us to identify the WM microstructural features of early PD without being confounded by various disease staging and medications effects. How PD pathology affects the alterations of WM at different disease stages would nonetheless require longitudinal follow-up, which is a limitation of our current study.

In conclusion, in this large cohort of newly diagnosed PD patients, we demonstrated increased FA and decreased MD and RD in several callosal, projection, and association tracts, and an inverse correlation between FA and motor severity but positive correlations between MD and RD and motor severity in patients with PD. Findings from connectometry analysis support enhancement of the WM connectivity in early disease stages. These DTI-derived alterations can be seen as a manifestation of neural compensation in the early disease stage. Longitudinal follow-up studies are warranted to identify the trajectory of WM changes alongside the progression of PD.

## Methods

### Participants

All participants in the current study were obtained from the Parkinson’s Progression Markers Initiative (PPMI, http://www.ppmi-info.org/). The PPMI is an observational, international, multi-centre study designed to identify PD progression biomarkers. The study was approved by the institutional review board of all participating sites in the Europe, including Attikon University Hospital (Greece), Hospital Clinic de Barcelona and Hospital Universitario Donostia (Spain), Innsbruck University (Austria), Paracelsus-Elena Clinic Kassel/University of Marburg (Germany), Imperial College London (UK), Pitié-Salpêtrière Hospital (France), University of Salerno (Italy), and in the USA, including Emory University, Johns Hopkins University, University of Alabama at Birmingham, PD and Movement Disorders Center of Boca Raton, Boston University, Northwestern University, University of Cincinnati, Cleveland Clinic Foundation, Baylor College of Medicine, Institute for Neurodegenerative Disorders, Columbia University Medical Center, Beth Israel Medical Center, University of Pennsylvania, Oregon Health & Science University, University of Rochester, University of California at San Diego, University of California, San Francisco. Written informed consent was obtained from all participants before study enrolment. The study was performed in accordance with relevant guidelines and regulations. To be enrolled into the PPMI study, all patients were required to fulfill the following criteria: 1) met the standard diagnostic criteria for PD, 2) diagnosed within 2 years before the initial visit, 3) Hoehn & Yahr (H&Y) stage ≤ 2 at baseline, 4) demonstrated deficits of dopamine transporters (DATs) on single-photon emission CT (SPECT) imaging, and 5) not on any PD medication at baseline. All HCs were required to have normal DATs and be free of any significant neurological disorders and medications that might interfere with the results of DAT SPECT imaging. All participants received comprehensive clinical assessments of motor, cognitive and behavioral functions at study entry. Only participants (HCs and PDs) with baseline DTI and with minimal head motion (i.e., translation: ≤3 mm; rotation: ≤2 degrees) during DTI acquisition were included in the current study.

### Clinical Assessment

After initial screening, all participants were comprehensively assessed at the baseline visit for clinical performance on motor, non-motor, cognitive, and neuropsychiatric functions by the site investigators. Specifically, motor severity was evaluated using UPDRS-III^16^. Global cognitive function was assessed using the Montreal Cognitive Assessment (MOCA)^33^. Neuropsychiatric assessment was performed using the 15-item Geriatric Depression Scale (GDS)^34^.

### MRI Acquisition

MRI was performed using a standardized protocol on 3T Siemens scanners (all Siemens Healthcare, Malvern, PA). Details of the MRI acquisition can be found in the MRI technical operations manual at http://www.ppmi-info.org/. In brief, for each patient, a 2-dimensional echo-planar DTI sequence was acquired for each participant using the following parameters: TR/TE = 900/88 ms, flip angle = 90°, voxel size = 2 × 2 × 2 mm^3^, 72 slices, 64 gradient directions with a b-value of 1000 s/mm^2^. One non-gradient volume (b = 0 s/mm^2^) was also acquired. The MRI protocol was distributed to each recruiting site to ensure consistent installations.

### DTI Preprocessing

All the DTI data were first converted from the Digital Imaging and Communications in Medicine (DICOM) format to the Neuroimaging Informatics Technology Initiative (NIfTI) format, and preprocessed using FSL 5.0.7 (http://fsl.fmrib.ox.ac.uk/fsl). Eddy currents and head motion were corrected by affine registration to the first b0 image using the ‘eddy_correct’ function in FSL. Each participant’s movement in x, y, and z coordinates was computed based on the output of eddy_correct. A brain mask was created using the fractional intensity threshold of 0.3 to ensure that only diffusion tensors inside the brain were computed. The diffusion tensors were then linearly fitted to the diffusion-weighted images using the ‘dtifit’ tool in FSL, generating maps of FA, MD, AD, and RD.

A measure of volume-to-volume displacement was obtained for each participant using the output file of the ‘eddy_correct’ motion correction function. To reduce the effect of excessive head motion during DTI acquisition, only participants who showed no more than 3-mm translational and 2-degree rotational movements were included in the study.

### Tract-Based Spatial Statistics (TBSS) Analysis

The TBSS tool in FSL^26^ was used to compare DTI measures between PD patients and HCs and to correlate significant DTI measures with motor dysfunction. First, all participants’ FA maps were nonlinearly aligned to the predefined FSL FMRIB58 FA map and registered to MNI152 standard space. The mean FA skeleton, a representation of the centre of the white matter tracts common to all subjects, was created and thresholded at FA > 0.20. The aligned FA map of each participant was projected onto the FA skeleton. A similar process was done for the MD, AD, and RD maps, such that without the initial registration, these diffusivity maps were projected onto the mean FA skeleton.

### Diffusion MRI Connectometry

Diffusion MRI connectometry was used to further explore group difference. The DTI data were reconstructed in a common stereotaxic space using q-space diffeomorphic reconstruction (QSDR)^27^ with a diffusion sampling length ratio of 1.25. The output resolution was 2 mm. Group comparisons were performed to determine difference between PD patients and HCs. Additionally, for the PD group, multiple regression was performed to determine local connectome associated with motor severity. A deterministic fibre tracking algorithm^13^,^35^ with a threshold of 0.05 (FDR correction for multiple comparisons) and at least 10% group difference was used to select local connectomes that showed significant difference or association with motor severity. To estimate the FDR, a total of 2000 randomised permutations were applied. The analysis was performed using the DSI Studio (http://dsi-studio.labsolver.org).

### Statistical Analysis

Demographical, clinical, neuropsychological data and head motion during DTI acquisition were analysed using IBM Statistical Package for Social Sciences software (version 21; SPSS, Inc., Chicago, IL). For continuous variables, one-way analysis of variance (ANOVA) and independent-sample t tests whenever appropriate were used to compare group differences. Pearson’s chi-squared or Fisher’s exact tests were used to compare categorical variables.

One-way ANOVA was performed in a general lineal model using ‘randomise’ in FSL to test group differences in FA, MD, RD, and AD between the HY1PD, HY2PD and HC groups. This program used permutation-based testing with 5000 permutations and statistical inference by applying threshold-free cluster enhancement (TFCE)^36^ with a threshold of P < 0.01, corrected for multiple comparisons. Any regions on the skeleton showing significant clusters were localised using the John Hopkins University ICBM-DTI-81 White Matter labels and John Hopkins University White Matter Tractography atlases in FSL. DTI information of tracts showing significant group differences were extracted and correlated with motor severity using Pearson’s correlation with significance being defined at p < 0.05 (Bonferroni corrected).

### Data Availability

Data used in the preparation of this article were obtained from the Parkinson’s Progression Markers Initiative data-base (www.ppmi-info.org/data). For up-to-date information on the study, visit www.ppmi-info.org.

## Additional Information

**How to cite this article**: Wen, M.-C. *et al*. White matter microstructural characteristics in newly diagnosed Parkinson’s disease: An unbiased whole-brain study. *Sci. Rep.* **6**, 35601; doi: 10.1038/srep35601 (2016).

## Figures and Tables

**Table 1:** Demographic and clinical characteristics.

	HC (N = 60)	HY1PD (N = 64)	HY2PD (N = 87)	p
	*Mean (SD*)	*Mean (SD*)	*Mean (SD*)	
Age (years)	60.33 (10.84)	60.16 (9.57)	62.01 (9.33)	0.44
Gender, male N (%)†	40 (66.7)	41 (64.1)	55 (63.2)	0.67
Education (years)	15.5 (2.87)	14.83 (3.26)	15.55 (3.06)	0.31
Handedness, R (%)†	75.0	89.1	87.4	0.20
PD duration (months)‡	—	5.63 (6.37)	7.55 (7.38)	0.11
UPDRS-III*	0.62 (1.40)	14.70 (5.68)	25.14 (8.61)	<0.001
MOCA	28.17 (1.14)	27.50 (2.38)	27.64 (1.92)	0.12
GDS	4.92 (1.00)	4.89 (1.05)	4.92 (0.92)	0.98
Head motion				
Translation (mm)	0.42 (0.18)	0.46 (0.22)	0.46 (0.19)	0.31
Rotation (degree)	0.18 (0.15)	0.21 (0.13)	0.19 (0.13)	0.42

Note: all analyses were one-way ANOVA, except ^†^using *χ*^2^ or Fisher’s Exact test and ^‡^using independent-sample t test. *Post-hoc analysis indicated that significant differences between HC and HY1PD, HC and HY2PD, and HY1PD and HY2PD groups. Handedness R = right-handedness; UPDRS-III = MDS-Unified Parkinson’s Disease Rating Scale-Motor Subscale; H&Y = Hoehn & Yahr staging; MOCA = Montreal Cognitive Assessment; GDS = Geriatric Depression Scale.
